# Strong phylogenetic signals and phylogenetic niche conservatism in ecophysiological traits across divergent lineages of Magnoliaceae

**DOI:** 10.1038/srep12246

**Published:** 2015-07-16

**Authors:** Hui Liu, Qiuyuan Xu, Pengcheng He, Louis S. Santiago, Keming Yang, Qing Ye

**Affiliations:** 1 Key Laboratory of Vegetation Restoration and Management of Degraded Ecosystems, South China Botanical Garden, Chinese Academy of Sciences, Xingke Road 723, Guangzhou 510650, China; 2 University of Chinese Academy of Sciences, Yuquan road 19A, Beijing 100049, China; 3 Botany & Plant Sciences, University of California, 2150 Batchelor Hall, Riverside, CA 92521-0124, USA; 4 Smithsonian Tropical Research Institute, P.O. Box 0843-03092, Balboa, Ancon, Panama, Republic of Panama; 5 Horticulture Center, South China Botanical Garden, Chinese Academy of Sciences, Tianyuan Road 1190, Guangzhou 510520, China

## Abstract

The early diverged Magnoliaceae shows a historical temperate-tropical distribution among lineages indicating divergent evolution, yet which ecophysiological traits are phylogenetically conserved, and whether these traits are involved in correlated evolution remain unclear. Integrating phylogeny and 20 ecophysiological traits of 27 species, from the four largest sections of Magnoliaceae, we tested the phylogenetic signals of these traits and the correlated evolution between trait pairs. Phylogenetic niche conservatism (PNC) in water-conducting and nutrient-use related traits was identified, and correlated evolution of several key functional traits was demonstrated. Among the three evergreen sections of tropical origin, *Gwillimia* had the lowest hydraulic-photosynthetic capacity and the highest drought tolerance compared with *Manglietia* and *Michelia*. Contrastingly, the temperate centred deciduous section, *Yulania*, showed high rates of hydraulic conductivity and photosynthesis at the cost of drought tolerance. This study elucidated the regulation of hydraulic and photosynthetic processes in the temperate-tropical adaptations for Magnoliaceae species, which led to strong phylogenetic signals and PNC in ecophysiological traits across divergent lineages of Magnoliaceae.

Divergent evolution among closely related species has received great attention in the study of species distribution and coexistence^1^,^2^. Because closely related species share more common evolutionary history than distantly related species, they tend to have similar niche-related traits, a pattern known as phylogenetic niche conservatism (PNC)^3^,^4^,^5^. However, during adaptation to variable environmental conditions, certain ecophysiological traits could also be labile causing trait divergences within a lineage^3^. Various phylogenetic models have been built to detect patterns (conserved, random or convergent traits), rates (slow or rapid evolution) and modes (gradual or punctuated evolution) of trait evolution^4^,^6^,^7^, with subtly different assumptions. For example, under a Brownian motion (BM) model of trait evolution, PNC arises as species inherit their niches from ancestors, but then slowly diverge during movement into new habitats^7^; whereas under an Ornstein-Uhlenbeck (OU) model, niches of species are constrained, so that stabilizing selection prevents species from moving too far from the optimum niche^8^, and phylogenetic signals are weaker than predicted by a BM model. Therefore, without clear assumptions of a specific trait evolution model, it is difficult to infer the underlying evolutionary processes through analysis of phylogenetic signal^5^,^6^,^9^.

Contrary to the slow divergences indicated by PNC, adaptive radiation implies rapid divergence, which could be distinguished by rates of trait evolution^10^. Although clades with lower evolutionary rates will have more conserved niches than clades with higher evolutionary rates, calculating evolutionary rates alone can not test whether niches are conserved, but provides comparisons of the degree of PNC among lineages^6^. The relationship between process, rate and phylogenetic signal is complex^9^,^10^, thus careful model selections are essential to integrate phylogenetic comparative methods with experimental data to test hypotheses in evolutionary ecology such as trait adaptation and correlated evolution^11^.

Many phylogenetic conservative ecophysiological traits among closely related species have been identified, indicating their similar responses to environmental changes^12^. However, contrasting life form, height, transpiration and relative growth rates have also been detected for closely related species such as within a genus^13^,^14^. In addition, convergent hydraulic architecture and drought resistance have been shown among distantly related phylogenetic clades such as in different plant families^15^,^16^. Hence, phylogenetic relatedness is essential in comparative studies focusing on the evolutionary patterns of a single trait, as well as the correlated evolution of trait pairs^5^,^17^.

Plants adjust their ecophysiological traits to compete for resources in various environments, with trade-offs between co-adapted traits^18^. For example, deciduous species tend to exhibit higher specific leaf area (SLA), nutrient content, photosynthetic capacity and growth rate than evergreens at a cost of leaf longevity^19^,^20^,^21^. There are also strong selections on canopy species to grow tall and compete for light, but tree heights could be constrained by hydraulic limits^22^,^23^. Under contrasting moisture conditions, the plant water transport system can sometimes show a trade-off between efficiency (hydraulic conductivity) and safety (vulnerability to cavitation)^24^,^25^. Moreover, plant drought tolerance/resistance also differs widely, with species occurring in drier habitats usually displaying lower leaf turgor loss point (*Ψ*_tlp_)^26^. Furthermore, suites of ecophysiological traits can interact with each other such as hydraulic-photosynthetic coordination^27^,^28^. Hence, a variety of plant traits that represent several axes of ecological strategy variation should be analysed for a sound understanding of which ecological mechanisms determine the trajectories of adaptation.

As an early diverged angiosperm family, Magnoliaceae is extraordinary important for studying the evolution of flowering plants from phylogenetic and geographical perspectives^29^,^30^,^31^,^32^. Nearly 80% of the over 200 Magnoliaceae species are currently distributed in eastern to south-eastern Asia, especially in South China; the other 20% are distributed in North and South America^33^,^34^. Previous phylogenetic work suggested that the divergence time between two closely related lineages of Magnoliaceae separating into temperate (*Yulania*) and tropical (*Michelia*) areas was before the Oligocene^30^,^34^. Yet, whether different ecophysiological traits have been labile during the evolution of this family remains unclear.

According to the taxonomic classification of Figlar & Nooteboom (2004)^35^ and the records of Magnolias in China^33^, one the four largest Magnoliaceae lineages is centred in temperate China (subgenus *Yulania* section *Yulania*, deciduous trees/shrubs), whereas three are centred in tropical China (subgenus *Yulania* section *Michelia*, evergreen trees/shrubs; subgenus *Magnolia* section *Gwillimia*, evergreen shrubs; and subgenus *Magnolia* section *Manglietia*, evergreen trees). In this study, 27 Magnoliaceae species from the four sections conserved in South China Botanical Garden were selected. Considering the temperate-tropical distributions, we measured 20 ecophysiological traits related to resource use (mainly hydraulics and photosynthesis), and tested their phylogenetic signals and correlated evolution between trait pairs. We hypothesized that these ecophysiological traits might be phylogenetically conserved and correlated, which could be explained by the divergent evolution of Magnoliaceae.

## Results

### Phylogenetic signals and trait divergences of the four Magnoliaceae sections

The majority of plant traits tested in this study showed strong phylogenetic signals based on Pagel’s *λ* (*λ* > 0.50 and *P* < 0.05 for *λ* = 0), except *Ψ*_tlp_, *A*_area_, *g*_s_, *E*, WUE_i_, leaf P and N/P; and Blomberg’s *K* of each trait showed congruent patterns as Pagel’s *λ*, except the lower absolute magnitude of *K* (Table 1). The natural environmental variables for each species all showed low phylogenetic signals. Consistency of the results from 300 phylogenetic trees was confirmed by histograms of *K* and *λ* values (Supplementary Fig. S1).

**Figure 1:**
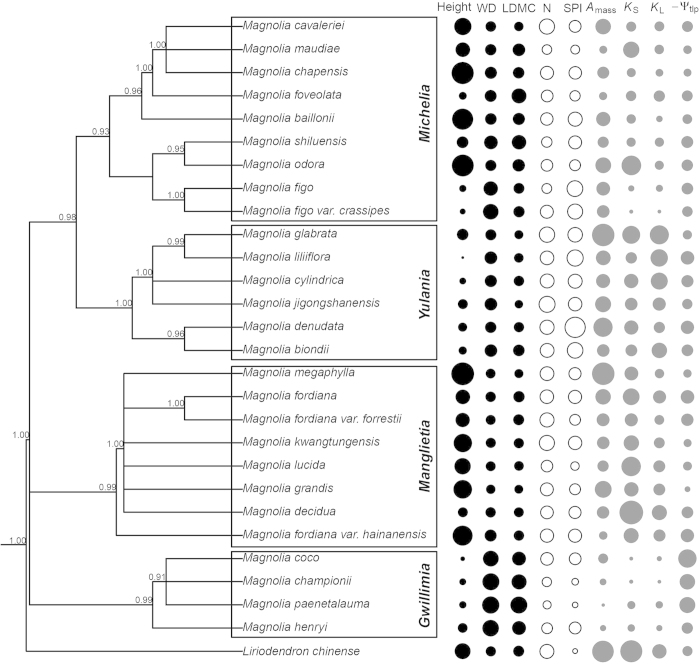
Phylogenetically dependent ecophysiological traits mapped across a phylogenetic tree of 27 Magnoliaceae species. The phylogenetic tree was constructed based on the *matK* and *trnH* sequences and rooted by defining *Liriodendron chinense* as the sister group to all other Magnoliaceae species. Posterior probabilities are reported above each node, the four sections are indicated by boxes. Trait values in each column are in proportion to the size of circles for each species, where larger circles indicate higher values, but the original values of *Ψ*_tlp_ are negative so we used −*Ψ*_tlp_ for plotting. Black columns indicate plant height, WD and LDMC, white columns are leaf N concentration and SPI, and grey columns indicate *A*_mass_, *K*_*S*_, *K*_L_ and −*Ψ*_tlp_. See Table 1 for trait abbreviations and Supplementary Table S3 for their original values.

Plant traits showed clear divergences among the four largest sections in Magnoliaceae (Fig. 1). The strict consensus tree based on multiple DNA sequences verified sect. *Yulania* and sect. *Michelia* were closely related (posterior probability = 0.98), but the relationships among this clade and the two other clades remain unresolved. Big and tall trees existed in sect. *Michelia* and *Manglietia*, while high WD and high LDMC occurred in sect. *Michelia* and *Gwillimia*. Species in sect. *Yulania* showed higher leaf N and SPI than the others. Higher photosynthesis, hydraulic conductivity and *Ψ*_tlp_ were found for species in sect. *Yulania* and *Manglietia* (Fig. 1; Supplementary Table S3).

### Phylogenetic principal component analysis (PPCA) results

**Figure 2:**
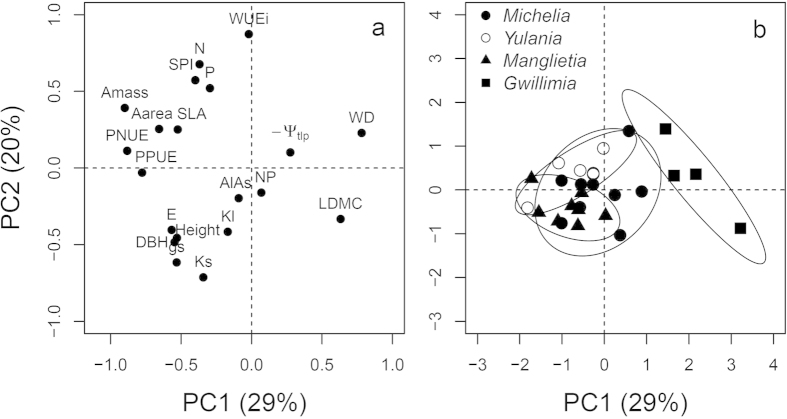
Phylogenetic principal component analysis (PPCA) for the first two principal components (PC) of 27 Magnoliaceae species. (**a**) PC loadings and (**b**) species scores with four sections circled as *Yulania* (white dots), *Michelia* (black dots), *Gwillimia* (black squares) and *Manglietia* (black triangles). The percentages of variance explained by the first two PCs are in the axis labels. See Table 1 for trait abbreviations and Supplementary Table S4 for values of PC loadings.

Against the phylogenetic background, PPCA for the 20 ecophysiological traits showed that the first two axes explained 29% and 20% of total variation, respectively (Fig. 2; Supplementary Table S4). Photosynthetic related traits (*A*_mass_, *A*_area_, PNUE and PPUE) clustered together on the negative side of PC1, while WD and LDMC were loaded on the positive side of PC1. Hydraulic related traits (*K*_S_ and *K*_L_) and WUE_i_ were loaded on the negative and positive side of PC2, respectively. All other traits were unrelated with the first two PCs (Fig. 2a). Sect. *Gwillimia* could be distinguished from other sections along PC1, but sect. *Yulania*, *Michelia* and *Manglietia* could not be separated (Fig. 2b).

### Phylogenetic correlations between ecophysiological traits

**Figure 3:**
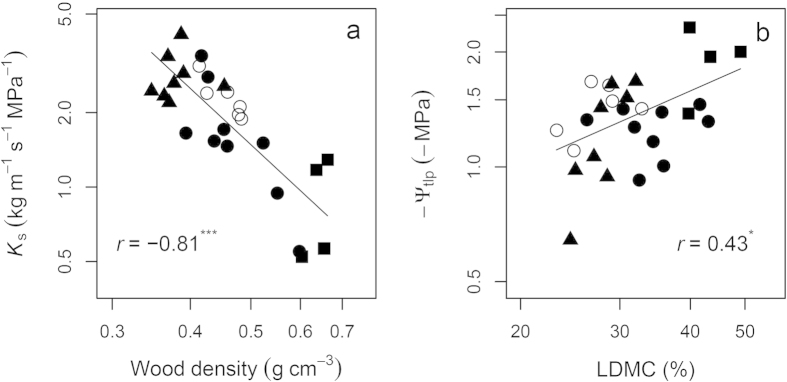
Correlations between (a) *K*_S_ and sapwood density, (b) *Ψ*_tlp_ and LDMC for 27 Magnoliaceae species. Figures are plotted on logarithmic scales and models are also fitted on logged mean species values. Because the original values of *Ψ*_tlp_ are negative, log(−*Ψ*_tlp_) are used in the models. Correlations are based on the phylogenetic generalized linear model (*PGLM*, solid line), with correlation coefficients *r* and *P* values for each model. The four sections are *Yulania* (white dots), *Michelia* (black dots), *Gwillimia* (black squares) and *Manglietia* (black triangles).

**Figure 4:**
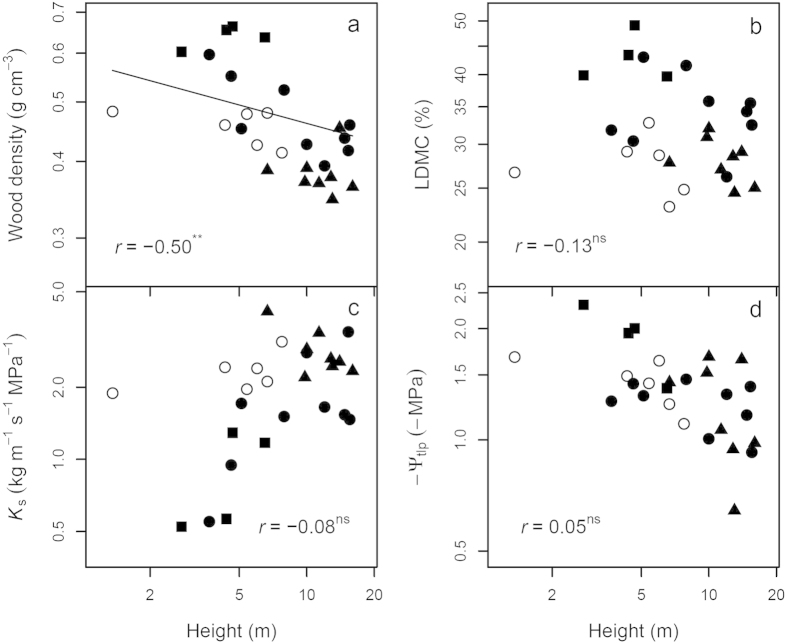
Correlations between plant height and sapwood density, LDMC, *K*_S_ and *Ψ*_tlp_ for 27 Magnoliaceae species. In d, −*Ψ*_tlp_ are used in models due to the original negative values of *Ψ*_tlp_. For all figures, symbols for the four sections, models, fitted lines, correlation coefficients and *P* values are the same as in Fig. 3.

*K*_S_ showed a strong negative relationship with WD at the branch level, while −*Ψ*_tlp_ displayed a positive relationship with LDMC at the leaf level, but neither correlation was affected by phylogeny (Fig. 3). WD, *K*_S_ and −*Ψ*_tlp_ were also correlated with height, but such relationships became significantly weaker (WD) or even vanished (*K*_S_ and −*Ψ*_tlp_) if phylogeny was taken into account (Fig. 4).

**Figure 5:**
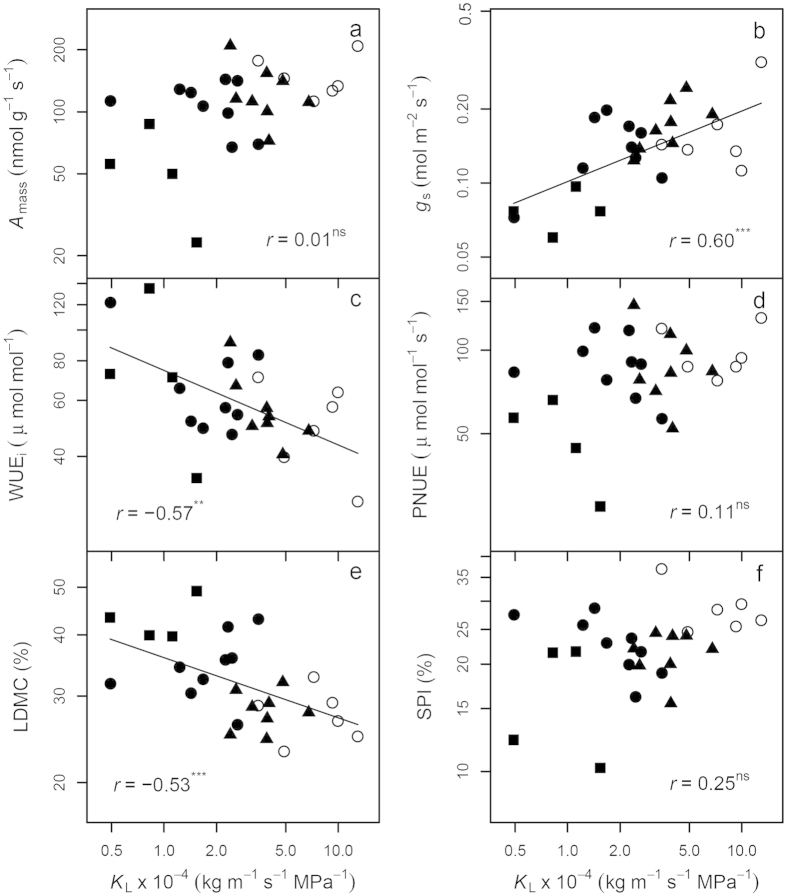
Correlations between leaf specific hydraulic conductivity (*K*_L_) and plant traits for 27 Magnoliaceae species. (**a**–**d**), photosynthesis related traits; (**e**–**f**), leaf structural traits (LDMC and SPI). For all figures, symbols for the four sections, models, fitted lines, correlation coefficients and *P* values are the same as in Fig. 3.

As a measure of the hydraulic efficiency of the stem to supply water to distal leaves, *K*_L_ showed positive correlations with *A*_mass_ and *g*_s_, and a negative relationship with WUE_i_ (Fig. 5a–c). Although there were positive trends for *K*_L_ in relation to PNUE and SPI, correlations were not statistically significant. *K*_L_ was negatively correlated with LDMC (Fig. 5d–f).

## Discussion

The great geographical and ecophysiological divergences among Magnoliaceae lineages may explain the strong phylogenetic signals in a suite of ecophysiological traits measured in this study (Fig. 1, Table 1). Under a BM model of trait evolution, we interpreted strong phylogenetic signals (traits with *λ* > 0.50, *P *< 0.05 for *λ *= 0) in ecophysiological traits as evidence of PNC^6^,^36^. As *λ* generally outperforms *K* in detecting phylogenetic signals, and *K* is suitable for models with changing evolutionary rates^37^, we used *λ* values to judge phylogenetic signals in each trait. The congruence of phylogenetic patterns based on *K* and *λ* values further supported our interpretations of the *λ* results. Overall, plant height, DBH, WD, LDMC, SPI, SLA, *A*_L_/*A*_S_, *K*_S_ and *K*_L_, as well as *A*_mass_, PNUE and PPUE were all phylogenetically conservative, consistent with a number of previous studies showing strong phylogenetic signals in similar ecophysiological traits^13^,^14^,^38^. Although PNC is associated with different evolutionary processes^4^, such as genetic drift, stabilizing selection^8^, linkage of co-adapted traits, or restricted genetic variation during evolution^3^. We found in our results the conserved traits were closely related to plant hydraulics and nutrient use, which might be caused by stabilizing selection of water and nutrient availability in different habitats and correlated adaptations between structural and functional traits during evolution. By contrast, we found some photosynthetic traits (*A*_area_, *g*_s_, *E* and WUE_i_) were highly labile and phylogenetically independent (Table 1), which might be due to the quick responses and convergent evolution of these traits to environmental changes^39^.

Magnoliaceae species originated ~90 million years ago (mya)^29^ and started the complicated divergent evolution ~55 mya^34^, but the absolute evolutionary rate of Magnoliaceae was unclear in our study due to the lack of a time-calibrated phylogeny. Although the relationship between phylogenetic signals and evolutionary rate is complex^9^, the relatively low *K* values of the measured ecophysiological traits (Table 1) might indicate an early adaptive radiation similar to the examples in Ackerly (2009). *K* < 1 has been consistently reported based on experimental data^10^,^40^, potentially due to strong adaptive evolution or even stabilizing selection under an OU model^10^. The processes of stabilizing selection and correlated adaptation were also inferred from *λ* values (Table 1).

Our results of ecophysiological traits correspond to patterns of divergent evolution, along distribution (tropical *vs.* temperate area), leaf form (evergreen *vs.* deciduous) and growth form (tree *vs.* shrub), among the four largest sections of Magnoliaceae (Figs 1 and 2; Supplementary Table S3). Leaves of deciduous species usually exhibit higher nutrient concentration and greater SLA than co-occurring evergreen species^19^,^20^,^21^, whereas tough leaves with stiff leaf cell walls and low *Ψ*_tlp_ are features of evergreen leaves in relatively arid regions^26^. This pattern describes the mainly deciduous *Yulania* species centred in temperate areas, which displayed the highest leaf N, leaf P, SLA and SPI, and the lowest LDMC, WD and *A*_L_/*A*_S_, showing more drought sensitivity than other species. Although *Yulania* was separated from its closely related evergreen *Michelia* early before the Oligocene^34^, hydraulic and photosynthetic traits (*K*_S_, *A*_area_ and *A*_mass_) of *Yulania* were similar to *Michelia* but lower than *Manglietia*. Meanwhile, *Manglietia* is a basal lineage in Magnoliaceae with primitive reproductive traits, and has remained in tropical forests as evergreen trees, whereas *Gwillimia* has evolved independently as understory evergreen shrubs in tropical or subtropical forests^33^.

Although some species have been reported to have greater vulnerability to drought induced cavitation and higher gas exchange rates when growing in wet habitat^27^,^28^, this is not the case for sect. *Gwillimia* in this study. The tropical-originated *Gwillimia* species were all drought tolerant evergreen shrubs with the lowest hydraulic conductivity, photosynthetic rate based on either leaf biomass (*A*_mass_) or nutrient content (PNUE and PPUE), and the highest WUE_i_ and leaf drought tolerance (lowest *Ψ*_tlp_ values) among the four studied sections. It was reasonable that shrubs had lower hydraulic conductivity than trees or lianas to potentially cope with shade or otherwise low resource environments, which could be accomplished by narrower vessels^41^, more complex branching systems and more hydraulic constrictions caused by branch junctions^42^.

Phylogenetic signals in plant traits also indicated phylogenetic influences on trait correlations. Indeed, we found that most of the trait correlations were the products of coordinated evolution (Figs 2, 3, 4, 5)^13^,^36^. This might be due to greater divergences in deep nodes of the phylogenetic tree compared to descendent lineages (family or genus), which can frequently be the case under the BM model^40^. In some cases such as when plotting height against *K*s and −*Ψ*_tlp_ (Fig. 4c,d), or plotting *K*_L_ against *A*_mass_ (Fig. 5a), phylogenetic correlations were found to be close to zero, implying strong phylogenetic influences on height, *K*s and *K*_L_. Hence PNC in ecophysiological traits especially hydraulic traits, appear to be the basis for correlated trait evolution in Magnoliaceae^13^,^14^.

Hydraulic-photosynthetic coordination and a trade-off between water transport efficiency and drought tolerance were identified in Magnoliaceae species (Figs 3, 4, 5), as found in a number of previous studies^28^. The relationships of *K*_L_ with *A*_area_ and PNUE were not significant (Fig. 5d), probably due to the large variations in leaf N content among the four sections, which in turn affected *A*_area_ and PNUE^43^. As an essential index for plant size and biomass, we expected plant height to be correlated with numerous ecophysiological traits in terms of water transport and light interception^23^,^42^. Although significant correlations between height and a number of tested traits (*A*_L_/*A*_S_, single leaf area and dry weight, *g*_s_, WD, LDMC, *K*s and *Ψ*_tlp_) were identified, plant height was unexpectedly positively correlated with *K*s and *Ψ*_tlp_ (a negative correlation between height and −*Ψ*_tlp_ in Fig. 4d), which was seemly contrary to the theory of hydraulic limits to tree height^22^,^23^. In principal, water in tall trees experiences a greater tension and more resistance compared with short ones under similar moisture conditions, sometimes resulting in a lower hydraulic conductivity^44^,^45^. This was not the case in this study, as shrubs (all *Gwillimia* species, and some species of *Michelia* and *Yulania*) consistently displayed lower *K*s and *Ψ*_tlp_ (greater drought tolerance) than trees (all *Manglietia* species, and some species of *Michelia* and *Yulania*), consistent with the greatest water transport capacity where light availability is highest. Since most shrub species in this study are understory plants in tropical or subtropical forests with abundant water supply in their natural habitats (Supplementary Fig. S2)^33^, the low photosynthetic rate, *K*s and *Ψ*_tlp_ of shrubs could only be explained by the intrinsic differences between shrubs and trees. For example, more branch junctions in shrubs might cause greater hydraulic constrictions^42^, higher WD, narrower vessels and greater LDMC in shorter trees or shrubs might also contribute to the observed lower *K*s and *Ψ*_tlp_ in Magnoliaceae species as found in other species^23^,^25^,^26^.

It should be noted that common-garden experiments have been widely used to control environmental influences on traits^11^,^46^, and environmental variability did show strong effects on some ecophysiological traits like photosynthetic rate^47^, but for other traits such as cavitation resistance, the influence was limited^48^. We are aware that the majority of the 27 studied Magnoliaceae species are endemic in China, and the sampled individuals were transplanted from different parts of the county^49^. Hence, we compared mean annual temperature (MAT) and mean annual precipitation (MAP) at our study site with those of species’ naturally distributed areas, and found that our study site had higher MAT and MAP than that of many species’ original regions (Supplementary Fig. S2). Considering different degrees of phenotypic plasticity of different traits in response to environmental variability that might potentially bias the results, we agreed that an investigation on the effects of habitat variation on traits is needed in future work. Nevertheless, it has been shown that species grown in the same habitat like a common-garden tending to develop somehow convergent traits^15^,^16^. If this is the case in our study, one would expect weak phylogenetic signals of traits among species. However, strong phylogenetic signals had been detected, indicating that the “real” phylogenetic signals might be stronger. Such trait convergence in Magnoliaceae species would need to be verified by comparing traits measured in original habitats with traits measured in a common garden.

In conclusion, the phylogenetic signals detected in key functional traits, and the correlated evolution identified between trait pairs illustrated ecophysiological divergences in the four major sections of Magnoliaceae. Among the three tropical originated sections, *Gwillimia* species (evergreen shrubs) had the lowest hydraulic conductivity (*K*_S_ and *K*_L_), photosynthetic rate (*A*_area_ and *A*_mass_) and the highest drought tolerance (lowest *Ψ*_tlp_), whereas *Manglietia* species (evergreen trees) displayed the highest hydraulic and photosynthetic traits and the lowest drought tolerance. Centred in the temperate region, the mainly deciduous trees of *Yulania* showed high values of hydraulic and photosynthetic related traits at the cost of drought tolerance. Overall, our results elucidated hydraulic and photosynthetic regulation in the temperate-tropical adaptations for Magnoliaceae species, and revealed PNC in ecophysiological traits across divergent lineages of Magnoliaceae.

## Methods

### Study site and species sampling

Experiments were carried out in South China Botanical Garden (SCBG) (23°11’N, 113°21’E, 100 m altitude), the Chinese Academy of Sciences, Guangzhou, China, located in the south-subtropical monsoon climatic region. MAT is 21.2 °C, with 13.6 °C in January and 28.9 °C in July. MAP is ~1700 mm, of which 80% occurs in wet season from April to September. The World Magnolia Centre within SCBG is the largest conservation centre for magnoliaceous germplasm in the world and contains *~*150 Magnoliaceae species (Cultivated Flora of China, http://gardenflora.scbg.ac.cn/). In this study, 27 species from the four largest sections were selected (Supplementary Table S1). All sampled individuals were mature and grown in similar environmental conditions at SCBG^49^. Plant height and diameter at breast height (DBH, cm) were recorded for each individual.

### Phylogenetic tree

A phylogenetic tree of the 27 species was constructed based on two commonly sequenced chloroplast gene regions: *matK* and *trnH*. DNA sequences were retrieved from the GenBank of NCBI^50^ for 21 species and were isolated from fresh leaves for the other six species. The two markers were amplified by polymerase chain reaction (PCR) with primers published in literature (Supplementary Table S2). The sequences of the two genes were aligned using ClustalW^51^, followed by manual adjustments in BioEdit. Phylogenetic trees were built through Bayesian inference as implemented in MrBayes 3.2^52^, with the substitution model based on the corrected Akaike Information Criterion (AICc) values from jModelTest2^53^. The GTR + I + Γ model (a General Time Reversible model with a proportion of invariable sites and a gamma-shaped distribution of rates across sites) was selected as the optimal model. We did two independent runs for 10,000,000 generations each, sampled every 1,000 generation after a burn-in period of 4,000,000 to ensure stability and confirmed the convergence of two independent runs to the stationary distribution. A strict consensus tree was computed on the remaining sample trees pooled from the two independent runs, which was rooted by defining *Liriodendron* as the sister group to all other species of Magnoliaceae (Fig. 1). The relationships among species in the phylogenetic tree were verified with their closely related species in the same section in the latest phylogenies^31^. Although statistical comparative tests showed that polytomies and missing branch length information had negligible impacts on phylogenetic signals^37^, we ran phylogenetic sensitivity analyses to avoid potential biases. We randomly sampled 300 alternative phylogenies, and all phylogenetic tests were run across the 300 trees, with mean ± s.e.m. and histograms for each index reported.

### Hydraulic conductivity

Early in the morning, terminal branches (8 ~ 10 mm in diameter) from three to five individuals per species were excised using tree pruners. All stems were recut under water immediately and leaves were misted with water, and then samples were sealed in black plastic bags with moist towels to prevent transpiration and quickly transported to the laboratory. A stem segment 20 ~ 30 cm in length was cut from each branch under water with both cut ends trimmed using a razor blade. Branch segments were first flushed with degassed and filtered 20 mmol KCl solution at a pressure of 0.1 MPa for 10 min to remove air embolisms. Next a hydrostatic pressure generated by a 50 cm hydraulic head drove water flow through the segments. The downstream end of the segment was connected to a pipette and the time for fluid in the pipette to cross a certain graduation was recorded. Hydraulic conductivity (*K*_h,_ kg m s^−1^ MPa^−1^) equals the ratio of the water flux through the segment to the pressure gradient driving the flow. Sapwood specific hydraulic conductivity (*K*_S_, kg m^−1^ s^−1^ MPa^−1^) was calculated as *K*_h_ divided by the sapwood cross section area. Leaf specific hydraulic conductivity (*K*_L_, kg m^−1^ s^−1^ MPa^−1^) equals the ratio of *K*_h_ to the leaf area. The total leaf area attached to the stem segment (*A*_L_) was measured with a leaf area meter (Li-3000A; Li-Cor, Lincoln, NE, USA) to calculate the leaf to sapwood area (*A*_S_) ratio (*A*_L_/*A*_S_, m^2^ cm^−2^). Sapwood density (WD, g cm^−3^) was the ratio of dry mass to fresh volume from the same branches used to measure hydraulic conductivity. Sapwood samples with bark removed were saturated in water overnight. After wiping the surface, the sapwood volume was measured by the water displacement method, and was then oven-dried at 70 ^o^C for 72 h and weighed to obtain dry mass to calculate WD.

### Pressure volume curve

Leaf pressure volume curve analysis was based on the bench drying method^54^. Terminal branches from three to five individuals for each species were excised, recut underwater and rehydrated until leaf water potential was greater than −0.05 MPa. Leaf weight and water potential (*Ψ*_l_, measured by a pressure chamber; PMS, Corvallis, OR, USA) were measured periodically during desiccation. After all balanced pressure-weight measurements, leaves were oven-dried at 70 ^o^C for 72 h for dry weight and to calculate leaf dry matter content (LDMC, %). Leaf turgor loss point (*Ψ*_tlp_, MPa) was determined according to pressure volume relationship models^55^.

### Leaf gas exchange

Area-based photosynthetic rate (*A*_area_, μmol m^−2^ s^−1^), stomatal conductance (*g*_s_, mol m^−2^ s^−1^) and transpiration rate (*E*, mmol m^–2^ s^–1^) were measured between 9:00–11:00 with a portable photosynthesis system (Li-6400, LiCor, Lincoln, NE, USA). For tall trees, the sun-exposed branches were bent down to get access to living leaves. The photosynthetic photon flux density was set at 1500 μmol m^−2^ s^−1^ according to preliminary measurements, well above the levels at which most species saturate. Leaf temperature and chamber CO_2_ concentration were maintained at 28 °C and 400 ppm, respectively. Leaves were exposed to the above conditions for 5 minutes to allow the stabilization of photosynthetic parameters before recording. Three to five individuals were selected for each species and five leaves were measured for each individual. Intrinsic water use efficiency (WUE_i_, μmol mol^−1^) was calculated as *A*_area_/*g*_s_.

### Leaf structure, nutrients and stomatal pore area index (SPI)

Twenty fully expanded leaves from each individual were scanned by a leaf area meter, and then these leaves were oven-dried at 70 °C for 72 h for dry mass. Specific leaf area (SLA, cm^2^ g^−1^) was measured as leaf area divided by leaf dry mass, and leaf mass based photosynthetic rate (*A*_mass_, nmol g^−1^ s^−1^) was calculated as *A*_area_ × SLA. Dry leaves were ground and homogenized for leaf nutrient measurements. Total leaf nitrogen content (N, %) was determined by Kjeldahl analysis after digestion with concentrated H_2_SO_4_. Total phosphorus content (P, %) was analyzed by atomic absorption spectro photometry (UV-6000; Metash, Shanghai, China). Photosynthetic nitrogen and phosphorus use efficiencies (PNUE and PPUE) equalled *A*_area_/N and *A*_area_/P, respectively. Fresh leaves were used to make instant microscope slides using a sharp razor blade. Slides were observed under a microscope equipped with a digital camera (Optec, Chongqing Optec Instrument, China) and a computerized image analysis system (OPTPro2012 version 4.0, Optec software). Three epidermal peels from different leaves were measured for each species, and three images were randomly chosen as replicates for each peel. Guard cell length (GL) and width (GW) were measured, stomatal density (SD) was counted. The stomatal pore area index (SPI, %) measured stomata pore area per leaf area and was calculated as SD × GL^2^ 56.

### Data analysis and phylogenetic models

All data were analysed and figures were drawn in R 3.0.3^57^. Phylogenetic signals for all quantitative traits were calculated using both Blomberg’s *K*^58^ and Pagel’s *λ*^7^. *K* measures the extent to which a trait displays phylogenetic signal, where *K* = 0 indicates no phylogenetic signal, *K* = 1 suggests that the trait distribution perfectly conforms to Brownian Motion (BM), and *K* > 1 indicates stronger similarities among closely related species than expected under BM. Significance of *K* was evaluated based on comparison of the observed phylogenetic independent contrasts (PIC) and the expected contrasts under randomizations. A total of 999 randomizations were used to calculate *P* values of *K* based on variance of PICs. *K* was computed with function *phylosignal* in package *picante*^59^, which does not accept trees with polytomies, we thus randomly resolved the polytomies by transforming all multichotomies into a series of dichotomies (function *multi2di* in package *ape*^60^). Pagel’s *λ* measures correlations relative to the correlation expected under Brownian evolution^7^. It gives *λ* values between zero and one in which, *λ* = 0 indicates no phylogenetic signal and *λ* = 1 implies that the distribution of trait values across the phylogeny is exactly as expected under BM. We used the *pgls* function in the *caper* package^61^ to run *λ* tests to detect phylogenetic signal, and to calculate phylogeny corrected correlation coefficients for trait pairs accounting for variable levels of phylogenetic signal (phylogenetic generalized linear model, *PGLM*). Phylogenetic principal component analysis (PPCA) was employed to investigate key factors in distinguishing species with phylogeny taken into account^40^. Data were log-transformed to meet the requirement of normal distribution. If the variable had negative values such as leaf water potential, absolute values were used. PPCA was carried out using the *phyl.pca* function in R package *phytools*^62^. Differences in plant ecophysiological traits among the four sections of Magnoliaceae were tested by multiple comparisons (Tukey HSD).

## Additional Information

**How to cite this article**: Liu, H. *et al.* Strong phylogenetic signals and phylogenetic niche conservatism in ecophysiological traits across divergent lineages of Magnoliaceae. *Sci. Rep.* **5**, 12246; doi: 10.1038/srep12246 (2015).

## Supplementary Material

Supplementary Information

## Figures and Tables

**Table 1:** Phylogenetic signals for plant traits and environmental variables based on the phylogenetic tree of 27 Magnoliaceae species.

	**(a) Blomberg’s *K***		**(b) Pagel’s *λ***		
	***K***	***P*_(*rep *= *999*)_**	***λ***	***P*_(*λ *= 0)_**	***P*_(λ* *= 1)_**
Height (m)	0.23 ± 0.001	**	**0.58** ± **0.004**	*	**
DBH (cm)	0.28 ± 0.001	**	**0.72** ± **0.004**	**	0.13
WD (g cm^–3^)	0.66 ± 0.003	**	**0.92** ± **0.002**	***	0.09
*A*_L_/*A*_S_ (m^2^ cm^–2^)	0.25 ± 0.001	**	**0.65** ± **0.001**	**	**
*K*_S_ (kg m^–1^ s^–1^ MPa^–1^)	0.27 ± 0.001	**	**0.69** ± **0.004**	**	0.11
*K*_L_×10^–4^ (kg m^–1^ s^–1^ MPa^–1^)	0.39 ± 0.002	**	**0.84** ± **0.002**	***	0.05
SPI (%)	0.22 ± 0.002	*	**0.52** ± **0.003**	*	**
LDMC (%)	0.33 ± 0.003	**	**0.76** ± **0.005**	**	*
*Ψ*_tlp_ (MPa)	0.21 ± 0.003	0.28	0.51 ± 0.011	0.24	*
SLA (cm^2^ g^−1^)	0.24 ± 0.002	**	**0.59** ± **0.004**	*	**
*A*_area_ (μmol m^−2^ s^−1^)	0.18 ± 0.002	0.09	0.41 ± 0.003	0.19	**
*A*_mass_ (nmol g^−1^ s^−1^)	0.24 ± 0.002	**	**0.63** ± **0.003**	**	**
*g*_s_ (mol m^−2^ s^−1^)	0.20 ± 0.001	*	0.55 ± 0.005	0.06	**
*E* (mmol m^−2^ s^−1^)	0.14 ± 0.001	0.17	0.07 ± 0.003	0.72	***
WUE_i_ (μmol mol^−1^)	0.11 ± 0.001	0.29	0.00 ± 0.000	1.00	***
Leaf N (%)	0.17 ± 0.001	*	**0.52** ± **0.002**	*	***
Leaf P (%)	0.17 ± 0.002	**	0.44 ± 0.002	0.08	*
Leaf N/P	0.20 ± 0.003	**	0.45 ± 0.008	0.11	*
PNUE (μmol mol^–1^ s^–1^)	0.24 ± 0.003	**	**0.61** ± **0.004**	*	**
PPUE (mmol mol^–1^ s^–1^)	0.27 ± 0.004	**	**0.68** ± **0.006**	*	0.05
MAT_mean_ (°C)	0.20 ± 0.002	*	0.50 ± 0.004	0.11	**
MAT_min_ (°C)	0.19 ± 0.003	*	0.26 ± 0.005	0.31	*
MAT_max_ (°C)	0.19 ± 0.002	*	0.51 ± 0.005	0.20	**
MAT_range_ (°C)	0.13 ± 0.002	0.27	0.00 ± 0.000	1.00	**
MAP_mean_ (mm)	0.24 ± 0.002	**	0.69 ± 0.006	0.08	*
MAP_min_ (mm)	0.20 ± 0.002	*	0.49 ± 0.004	0.12	**
MAP_max_ (mm)	0.23 ± 0.002	0.10	0.04 ± 0.011	0.97	***
MAP_range_ (mm)	0.14 ± 0.002	0.37	0.00 ± 0.000	1.00	***

Results are (a) Blomberg’s *K* and (b) Pagel’s *λ* values, with mean ± s.e.m. and histograms for each index from the 300 phylogenetic trees reported (Supplementary Fig. S1). Sample sizes are 27, *P* values for *K*, *λ* = 0 and 1 are reported. Data for each trait were natural logged. Level of significance: *P < 0.05; **P < 0.01; ***P < 0.001. DBH, diameter at breast height; WD, sapwood density; *A*_L_/*A*_S,_ leaf to sapwood area ratio; *K*_S,_ sapwood specific hydraulic conductivity; *K*_L_, leaf specific hydraulic conductivity; SPI, stomatal pore area index; LDMC, leaf dry matter content; *Ψ*_tlp_ leaf turgor loss point; SLA, specific leaf area; *A*_area_, maximum CO_2_ assimilation rate per unit area; *A*_mass_, maximum CO_2_ assimilation rate per unit dry mass; *g*_s_, stomatal conductance; *E*, transpiration rate; WUE_i_, intrinsic water use efficiency; N, leaf nitrogen content; P, leaf phosphorus content; N/P, leaf nitrogen/phosphorus ratio; PNUE, photosynthetic nitrogen use efficiency; PPUE, photosynthetic phosphorus use efficiency; mean, minimum, maximum and range of mean annual temperature (MAT) and precipitation (MAP) for each species. Traits with *λ* values significantly different from zero are in bold.
