# Supplementary material for: Strong phylogenetic signals and phylogenetic niche conservatism in ecophysiological traits across divergent lineages of Magnoliaceae
Source: Sci Rep. 2015 Jul 16;5:12246. doi: 10.1038/srep12246 (PMC4503962; doi:10.1038/srep12246)
Supplement: Supplementary Information [file srep12246-s1.doc]

**Supplementary information**

**Strong phylogenetic signals and phylogenetic niche conservatism in ecophysiological traits across divergent lineages of Magnoliaceae**

Hui Liu, Qiuyuan Xu, Pengcheng He, Louis S. Santiago, Keming Yang and Qing Ye

**Table S1.** Species list with subgenus, section, life forms and accession numbers of DNA sequences from GenBank. The taxonomic system of Magnoliaceae follows Figlar & Nooteboom (2004).

| Species | Subgenus | Section | Growth form | Leaf form | *matK* | *trnH* |
| --- | --- | --- | --- | --- | --- | --- |
| *Liriodendron chinense* (Hemsl.) Sargent. | - | - | Tree | Deciduous | JN050093 | AB021046 |
| *Magnolia championii* (Bentham) N. H. Xia & C. Y. Wu | *Magnolia* | *Gwillimia* | Shrub | Evergreen | JN050033 | JN050197 |
| *Magnolia coco* (Loureiro) N. H. Xia & C. Y. Wu | *Magnolia* | *Gwillimia* | Shrub | Evergreen | JN050035 | AB021034 |
| *Magnolia henryi* (Dunn) N. H. Xia & C. Y. Wu | *Magnolia* | *Gwillimia* | Tree | Evergreen | JN050050 | AY009027 |
| *Magnolia paenetalauma* Dandy | *Magnolia* | *Gwillimia* | Tree | Evergreen | this study | this study |
| *Magnolia decidua* Q. Y. Zheng | *Magnolia* | *Manglietia* | Tree | Deciduous | AB055542 | AB055565 |
| *Magnolia fordiana* (Oliver) Hu var. *forrestii* (W. W. Smith ex Dandy) V. S. Kumar | *Magnolia* | *Manglietia* | Tree | Evergreen | this study | this study |
| *Magnolia grandis* (Hu & W. C. Cheng) V. S. Kumar | *Magnolia* | *Manglietia* | Tree | Evergreen | JN050048 | AY009022 |
| *Magnolia fordiana* var. *hainanensis* (Dandy) Nooteboom, comb. et stat. nov. | *Magnolia* | *Manglietia* | Tree | Evergreen | AF123478 | - |
| *Magnolia lucida* (B. L. Chen & S. C. Yang) V. S. Kumar | *Magnolia* | *Manglietia* | Tree | Evergreen | this study | this study |
| *Magnolia megaphylla* (Hu & W. C. Cheng) V. S. Kumar | *Magnolia* | *Manglietia* | Tree | Evergreen | this study | this study |
| *Magnolia kwangtungensis* Merrill | *Magnolia* | *Manglietia* | Tree | Evergreen | JN050052 | JN050170 |
| *Magnolia fordiana* (Oliver) Hu | *Magnolia* | *Manglietia* | Tree | Evergreen | this study | this study |
| *Magnolia baillonii* Pierre | *Yulania* | *Michelia* | Tree | Evergreen | - | AY009017 |
| *Magnolia cavaleriei* (Finet & Gagnepain) Figlar | *Yulania* | *Michelia* | Tree | Evergreen | JN050073 | AY009009 |
| *Magnolia chapensis* (Dandy) Sima | *Yulania* | *Michelia* | Tree | Evergreen | JN050074 | JN050193 |
| *Magnolia figo* var. *crassipes* (Y. W. Law) Figlar & Nooteboom | *Yulania* | *Michelia* | Shrub | Evergreen | JN050096 | JN050212 |
| *Magnolia figo* (Loureiro) Candolle | *Yulania* | *Michelia* | Shrub | Evergreen | JN050021 | AB021045 |
| *Magnolia foveolata* (Merrill ex Dandy) Figlar | *Yulania* | *Michelia* | Tree | Evergreen | JN050070 | AB623298 |
| *Magnolia maudiae* (Dunn) Figlar | *Yulania* | *Michelia* | Tree | Evergreen | JN050083 | HQ415444 |
| *Magnolia odora* (Chun) Figlar & Nooteboom | *Yulania* | *Michelia* | Tree | Evergreen | JN050056 | AY009013 |
| *Magnolia shiluensis* (Chun & Y. F. Wu) Figlar | *Yulania* | *Michelia* | Tree | Evergreen | JN050084 | JN050202 |
| *Magnolia biondii* Pampanini | *Yulania* | *Yulania* | Tree | Deciduous | JN050031 | - |
| *Magnolia cylindrica* E. H. Wilson | *Yulania* | *Yulania* | Tree | Deciduous | JN050067 | AY009020 |
| *Magnolia denudata* Desrousseaux in Lamarck | *Yulania* | *Yulania* | Tree | Deciduous | JN050037 | AB021037 |
| *Magnolia glabrata* Law et R.Z.Zhou ined. | *Yulania* | *Yulania* | Tree | Deciduous | this study | this study |
| *Magnolia jigongshanensis* T. B. Chao, D.L. Fu & W.B. Sun | *Yulania* | *Yulania* | Shrub | Deciduous | this study | this study |
| *Magnolia liliiflora* Desrousseaux in Lamarck | *Yulania* | *Yulania* | Shrub | Deciduous | JN050098 | JN050214 |

**Table S2.** Primers used for the amplifications of *matK* and *trnH* genes in this study.

| Primer | Sequence |
| --- | --- |
| *matK*_for | 5’-CGTACAGTACTTTTGTGTTTACGAG-3’ |
| *matK_*rev | 5’-ACCCAGTCCATCTGGAAATCTTGGTTC-3’ |
| *trnH*_for | 5’-CGCGCATGGTGGATTCACAATCC-3’ |
| *trnH*_rev | 5’-GTTATGCATGAACGTAATGCTC-3’ |

**Table S3.** Values and multiple comparison results of plant traits and environmental variables of the four main sections in Magnoliaceae. Data are mean±s.e.m. for species within each section (species numbers are given in brackets). There are 3~5 replicates for each species as described in Methods, and then mean values for each species are calculated, which are used for the multiple comparisons. Superscript letters are results of Tukey HSD. Lowest values with significant results in multiple comparative tests are in bold. See abbreviations in Table 1.

| Section | *Michelia* (9) | *Yulania* (6) | *Manglietia* (8) | *Gwillimia* (4) |
| --- | --- | --- | --- | --- |
| Height (m) | 9.9 ± 1.59 ^ab^ | 5.2 ± 0.91 ^a^ | 11.7 ± 1.02 ^b^ | **4.6 ± 0.77 ^a^** |
| DBH (cm) | 22.4 ± 3.62 ^b^ | 12.2 ± 1.74 ^ab^ | 23.1 ± 2.70 ^b^ | **6.2 ± 2.11 ^a^** |
| WD (g cm^–3^) | 0.5 ± 0.02 ^b^ | 0.5 ± 0.01 ^b^ | 0.4 ± 0.01 ^a^ | **0.6 ± 0.01 ^c^** |
| *A*_L_/*A*_S_ (m^2^ cm^–2^) | 0.9 ± 0.11 ^b^ | **0.4 ± 0.08 ^a^** | 0.8 ± 0.05 ^b^ | 0.9 ± 0.12 ^b^ |
| *K*_S_ (kg m^–1^ s^–1^ MPa^–1^) | 1.7 ± 0.29 ^ab^ | 2.3 ± 0.18 ^bc^ | 2.8 ± 0.23 ^c^ | **0.9 ± 0.20 ^a^** |
| *K*_L_×10^–4^ (kg m^–1^ s^–1^ MPa^–1^) | 2.0 ± 0.30 ^ab^ | 8.0 ± 1.42 ^c^ | 3.9 ± 0.49 ^b^ | **1.0 ± 0.22 ^a^** |
| SLA (cm^2^ g^–1^) | 126.8 ± 10.42 ^a^ | 192.9 ± 20.41 ^b^ | 135.9 ± 11.99 ^a^ | **94.1 ± 8.65 ^a^** |
| DMC (%) | 34.6 ± 1.76 ^b^ | **27.5 ± 1.40 ^a^** | 28.1 ± 0.92 ^a^ | 43.0 ± 2.19 ^c^ |
| SPI (%) | 22.8 ± 1.37 ^a^ | 28.6 ± 1.83 ^b^ | 21.5 ± 1.06 ^a^ | **16.4 ± 3.02 ^a^** |
| *Ψ*_tlp_ (MPa) | -1.3 ± 0.06 ^b^ | -1.4 ± 0.09 ^ab^ | -1.2 ± 0.13 ^b^ | **-1.9 ± 0.20 ^a^** |
| *A*_area_ (µmol m^–2^ s^–1^) | 8.7 ± 0.57 ^b^ | 8.0 ± 0.73 ^ab^ | 9.3 ± 0.53 ^b^ | **5.6 ± 1.13 ^a^** |
| *A*_mass_ (nmol g^-1^ s^-1^) | 109.1 ± 10.13 ^b^ | 150.8 ± 14.65 ^b^ | 127.0 ± 14.64 ^b^ | **54.1 ± 13.14 ^a^** |
| *g*_s_ (mol m^–2^ s^–1^) | 0.14 ± 0.01 ^b^ | 0.17 ± 0.03 ^b^ | 0.17 ± 0.01 ^b^ | **0.07 ± 0.01 ^a^** |
| E (mmol m^–2^ s^–1^) | 2.9 ± 0.31 ^a^ | 2.9 ± 0.44 ^a^ | 3.2 ± 0.19 ^a^ | 2.0 ± 0.11 ^a^ |
| WUE_i_ (µmol mol^–1^) | 67.8 ± 8.01 ^a^ | 51.4 ± 6.38 ^a^ | 57.2 ± 5.54 ^a^ | 78.2 ± 20.94 ^a^ |
| Leaf N (%) | 1.7 ± 0.09 ^ab^ | 2.1 ± 0.06 ^c^ | 2.0 ± 0.05 ^bc^ | **1.5 ± 0.14 ^a^** |
| Leaf P (%) | 0.10 ± 0.01 ^a^ | 0.15 ± 0.01 ^b^ | 0.10 ± 0.01 ^a^ | 0.10 ± 0.02 ^a^ |
| Leaf N/P | 18.4 ± 0.89 ^ab^ | 14.9 ± 1.12 ^a^ | 19.9 ± 1.13 ^b^ | 15.0 ± 1.38 ^a^ |
| PNUE (µmol mol^–1^ s^–1^) | 87.9 ± 7.65 ^b^ | 99.5 ± 8.54 ^b^ | 91.0 ± 10.13 ^b^ | **48.8 ± 8.42 ^a^** |
| PPUE (mmol mol^–1^ s^–1^) | 3.6 ± 0.38 ^b^ | 3.3 ± 0.45 ^ab^ | 4.0 ± 0.47 ^b^ | **1.6 ± 0.37 ^a^** |
| MAT_mean_ (°C) | 17.2 ± 1.03 ^ab^ | **14.0 ± 0.95 ^a^** | 17.8 ± 0.88 ^b^ | 19.5 ± 1.27 ^b^ |
| MAT_min_ (°C) | 12.3 ± 1.33 ^a^ | **8.8 ± 1.46 ^a^** | 14.1 ± 1.37 ^a^ | 15.2 ± 2.26 ^a^ |
| MAT_max_ (°C) | 21.6 ± 0.92 ^ab^ | **18.7 ± 0.72 ^a^** | 21.0 ± 0.91 ^ab^ | 23.7 ± 0.97 ^b^ |
| MAT_range_ (°C) | 9.2 ± 1.16 ^a^ | 9.9 ± 2.0 ^a^ | 7.0 ± 1.42 ^a^ | 8.5 ± 1.93 ^a^ |
| MAP_mean_ (mm) | 1457 ± 53 ^b^ | **1193 ± 62 ^a^** | 1422 ± 68 ^ab^ | 1494 ± 90 ^b^ |
| MAP_min_ (mm) | 1208 ± 69 ^b^ | **881 ± 81 ^a^** | 1231 ± 91 ^b^ | 1269 ± 119 ^b^ |
| MAP_max_ (mm) | 1755 ± 47 ^a^ | 1555 ± 96 ^a^ | 1653 ± 56 ^a^ | 1778 ± 119 ^a^ |
| MAP_range_ (mm) | 547 ± 82 ^a^ | 674 ± 121 ^a^ | 422 ± 88 ^a^ | 509 ± 133 ^a^ |

**Table S4.** Phylogenetic principal component analysis (PPCA) for the first two principal components based on 20 plant traits of 27 Magnoliaceae species. PC loadings and the percentage of variance explained by the first two PCs are reported. For each PC, the first six variables with highest loadings are in bold. See trait abbreviations in Table 1.

|  | PC1 | PC2 |
| --- | --- | --- |
| *A*_mass_ | **-0.90** | 0.39 |
| PNUE | **-0.88** | 0.11 |
| WD | **0.78** | 0.23 |
| PPUE | **-0.78** | -0.03 |
| *A*_area_ | **-0.66** | 0.25 |
| LDMC | **0.63** | -0.33 |
| WUE_i_ | -0.02 | **0.87** |
| *K*_S_ | -0.34 | **-0.71** |
| Leaf N | -0.37 | **0.68** |
| *g*_s_ | -0.53 | **-0.62** |
| SPI | -0.40 | **0.57** |
| Leaf P | -0.30 | **0.52** |
| Total variance % | 29% | 20% |
| Cumulative variance % | 29% | 49% |

**Figure S1.** Histograms of (a) Blomberg’s *K* and (b) Pagel’s *λ* values from 300 phylogenetic trees for indices in Table 1. *K* values are between 0 to 0.8, *λ* values are between 0 to 1.

**(a)**

**(b)**

**Figure S2.** Range of mean annual temperature (MAT, black bars) and precipitation (MAP, grey bars) for each species of the four Magnoliaceae sections in their naturally distributed areas in China. Horizontal lines are the means (solid lines) and standard deviations (dashed lines) for MAT (black lines) and MAP (grey lines) in the study site, respectively.

**Supplementary Methods for calculating climatic data in Figure S2.**

Geographical background included: (1) the administrative map of 348 municipalities of China, from National Geomatics Center of China (<http://ngcc.sbsm.gov.cn/>); (2) the altitude grid map of China at one square kilometre resolution, from DIVA-GIS (<http://www.diva-gis.org/>); (3) the grid maps of 1961-1999 MAT and MAP of China at one square kilometre resolution, from the Thematic Database for Human-earth System (<http://www.data.ac.cn/>).

The distribution of altitude range and municipalities for each species in China was recorded in “*Magnolias of China*” , the corresponding climatic data were calculated in three steps: (1) Extraction of municipalities for each species from administrative maps, followed by exaction of exact areas within municipalities based on altitudinal range. (2) Use of exact areas to mask and retrieve MAT/MAP maps, resulting in specific corresponding MAT/MAP data composed of 1 × 1 kilometre grid squares. (3) Summarizing of values of all grids for mean, maximum, minimum and range values for altitude, MAT and MAP. All processes were carried out in ArcGIS (version 9.3, Esri) for the 27 species.
